# Derivation of an Analytical Solution to a Reaction-Diffusion Model for Autocatalytic Degradation and Erosion in Polymer Microspheres

**DOI:** 10.1371/journal.pone.0135506

**Published:** 2015-08-18

**Authors:** Ashlee N. Ford Versypt, Paul D. Arendt, Daniel W. Pack, Richard D. Braatz

**Affiliations:** 1 Department of Chemical and Biomolecular Engineering, University of Illinois at Urbana-Champaign, Urbana, Illinois, United States of America; 2 Department of Chemical Engineering, Massachusetts Institute of Technology, Cambridge, Massachusetts, United States of America; 3 Department of Chemical and Materials Engineering, University of Kentucky, Lexington, Kentucky, United States of America; 4 Department of Pharmaceutical Sciences, University of Kentucky, Lexington, Kentucky, United States of America; Shanxi University, CHINA

## Abstract

A mathematical reaction-diffusion model is defined to describe the gradual decomposition of polymer microspheres composed of poly(D,L-lactic-co-glycolic acid) (PLGA) that are used for pharmaceutical drug delivery over extended periods of time. The partial differential equation (PDE) model treats simultaneous first-order generation due to chemical reaction and diffusion of reaction products in spherical geometry to capture the microsphere-size-dependent effects of autocatalysis on PLGA erosion that occurs when the microspheres are exposed to aqueous media such as biological fluids. The model is solved analytically for the concentration of the autocatalytic carboxylic acid end groups of the polymer chains that comprise the microspheres as a function of radial position and time. The analytical solution for the reaction and transport of the autocatalytic chemical species is useful for predicting the conditions under which drug release from PLGA microspheres transitions from diffusion-controlled to erosion-controlled release, for understanding the dynamic coupling between the PLGA degradation and erosion mechanisms, and for designing drug release particles. The model is the first to provide an analytical prediction for the dynamics and spatial heterogeneities of PLGA degradation and erosion within a spherical particle. The analytical solution is applicable to other spherical systems with simultaneous diffusive transport and first-order generation by reaction.

## Introduction

Poly(D,L-lactic-co-glycolic acid) (PLGA) microspheres are biodegradable polymeric devices that are widely studied for controlled-release drug delivery [1–4]. Compared to conventional drug dosage forms, controlled-release drug delivery can provide enhanced control of drug concentrations and biodistribution, reduce side effects, and improve patient compliance.

Drug molecules dispersed in the bulk polymer are released by diffusion by two main pathways: through the nondegraded polymer bulk and through aqueous pores that form in the polymer bulk as the polymer undergoes hydrolysis. Between microsphere size range extremes, drug release may transition from the diffusion-controlled regime (diffusive release through the nondegraded polymer bulk is faster through smaller microspheres that have shorter diffusion lengths than in larger microspheres) to the erosion-controlled regime (diffusive release through aqueous pores is faster through larger microspheres that have eroded porous interiors than in smaller microspheres). The drug release rates for drug molecules with different sizes and water solubilities depend strongly on the release regimes. Small, poorly water-soluble molecules are known to diffuse more easily through the nondegraded polymer bulk so are released more quickly in the diffusion-controlled regime [5, 6]. Conversely, small, highly water-soluble molecules and macromolecules are known to diffuse more easily through aqueous pores so are released more quickly in the erosion-controlled regime [7]. Thus, drug release is coupled to polymer degradation by the dynamics and spatial distribution of developing pores. To describe the development of the pore structure in the microspheres, it is first necessary to consider how the polymer reacts and forms pores.

PLGA degrades chemically by acid-catalyzed ester hydrolysis in the polymer bulk rather than from the surface inward because the water penetrates into the polymer matrix faster than the rate of hydrolysis [8]. The carboxylic acid end groups of the PLGA polymer chains can donate protons to autocatalyze the hydrolytic degradation, which accelerates the reaction kinetics. The amount of autocatalyst increases as polymer chains are broken to produce smaller chains because each of the smaller chains includes an autocatalytic end group. Small polymer fragments up to and including nonamers are water-soluble [9–11]. The water-soluble fragments dissolve in water and diffuse out of the polymer through water-filled pores, which leads to polymer erosion (mass loss) and increases pore volume in the microspheres. Autocatalytic hydrolysis is more substantial in the interior of large microspheres where the diffusion of degradation products is limited, leading to accumulation of acidic polymer end groups [7, 12–14].

Although many models have been proposed for drug release from PLGA microspheres [15–18], the mathematical formulation needed to accurately predict microsphere-size-dependent drug release is still unclear [18, 19]. Here, we focus on the clarifying the complex effects of autocatalysis on simultaneous polymer degradation and erosion in microspheres of different sizes. By considering the polymer in isolation without encapsulated drug molecules, we aim to contribute to the understanding of the development of porous domains in the microspheres, which can then be used incorporated into future models that include drug transport to describe the entire process of drug release from these types of dynamic porous materials.

The autocatalytic PLGA degradation reaction has been modeled previously [20, 21]; however, the models treated homogeneous degradation before the start of erosion, and the analytical solutions of the models do not depend on the microsphere size or spatial heterogeneities within the microspheres. Models using reaction-diffusion equations have been applied to a comparable drug delivery system: covalently bonded polymer-drug conjugates in solid polymers. The models included first-order cleavage of polymer-drug bonds followed by diffusive release of drug from polymer matrices [22, 23]. While the models for the polymer-drug conjugates treated a drug delivery system involving reaction and diffusion, the models cannot be applied directly to PLGA because the reaction kinetics in those models were independent of the drug diffusion dynamics. The reaction and diffusion processes for PLGA microspheres are coupled and should not be treated independently.

A reaction-diffusion model has been proposed previously [24] to treat autocatalytic degradation and erosion in the polymer plates and cylinders composed of chemically similar poly(lactic acid) (PLA), and the numerical predictions were compared to experimental data from a study on the degradation of PLA plates of different thicknesses [25]. While the model in [24] can be extended to other geometries using finite element analysis to solve the equations numerically, the influence of the relationship between diffusion and reaction parameters on the zones where diffusion and degradation have strong or weak influences was only presented for one-dimensional planar and cylindrical geometries. We aim to derive an analytical solution to a reaction-diffusion model specifically for the case of spherical geometry that can assess the relative dominance of the reaction and diffusion phenomena through a single dimensionless parameter.

Reaction-diffusion equations have commonly been used to model spherical catalyst pellets that experience simultaneous reaction and diffusion, and analytical solutions to the equations are available [26, 27]. Despite the similarities in the reaction-diffusion equations for the two systems, the catalyst pellets and PLGA microspheres differ in ways that are critical for their mathematical treatment: (1) the reaction term in PLGA is a generation term instead of a consumption term and (2) the directions of diffusion are reversed for PLGA microspheres and catalyst pellets. In catalyst pellets, the reactant diffuses into the sphere where it is consumed by a reaction. In contrast, the autocatalytic carboxylic acid end groups in PLGA microspheres are distributed throughout the sphere where more are generated by the degradation reaction, and some fraction of the autocatalyst diffuses out of the sphere. These differences between the physics of reaction and diffusion in spherical catalyst pellets and PLGA microspheres must be carefully accounted for during the analogous mathematical analyses of the reaction-diffusion equations used to model the systems.

Here, we derive an analytical expression for the transient, radial concentration of the autocatalytic carboxylic acid end groups of PLGA by simultaneously treating degradation and erosion of the polymer. Quantifying the transient, spatial distribution of the autocatalyst within the polymer is important for understanding how different conditions may contribute to accelerating hydrolysis in the interior of large microspheres and for preventing adverse effects of the acidic conditions within a microsphere on the drug stability or bioactivity [28]. Additionally, such a mathematical treatment provides insights into how the coupling between autocatalysis and drug diffusion may trigger transitions between diffusion-controlled and erosion-controlled drug release regimes.

## Methods

### Mathematical model for the autocatalyst concentration

The conservation equation for a chemical species subject to reaction and diffusion within a radially symmetric sphere is

$$\begin{array}{c} \frac{\partial c}{\partial t}=\frac{1}{r^{2}}\frac{\partial }{\partial r}(r^{2}\frac{D}{R^{2}}\frac{\partial c}{\partial r})+R_{V}, \end{array}$$ (1)

where *c*(*r*, *t*) is the concentration, 0 ≤ *r* ≤ 1 is the normalized radial position defined as $r=\hat{r}/R$, $\hat{r}$ is the the radial distance from the center of the sphere, *R* is the radius of the sphere, *t* ≥ 0 is time, *D* is the diffusion coefficient, and *R*_*V*_ is the net rate of generation of species per volume. We assume that the microsphere volume is constant for bulk-eroding PLGA, neglecting any effects of possible microsphere swelling. The chemical species of interest is the autocatalytic carboxylic acid end groups of the polymer chains or *autocatalyst*. PLGA degradation is often treated with pseudo-first-order kinetics [13, 18], where the autocatalyst undergoes first-order growth while the concentrations of water, *c*_H_2_O_ and ester bonds in the polymer, *c*_E_, are assumed to remain constant, giving

$$\begin{array}{c} R_{V}=kc, \end{array}$$ (2)

where the rate constant *k* for random ester bond hydrolysis incorporates the constant concentrations *c*_H_2_O_ and *c*_E_ (the applicability of the assumption of constant *c*_E_ is checked in the Results and discussion section). PLGA erosion is treated by assuming that all carboxylic acid end groups have a uniform constant diffusion coefficient *D* independent of the length of the polymer chain to which they are attached; with this assumption, diffusion of degradation products can be included in the analytical expression for autocatalyst concentration.

Substituting *α* = *D*/*R* ^2^ and (Eq 2) into (Eq 1) yields

$$\begin{array}{c} \frac{\partial c}{\partial t}=\frac{\alpha }{r^{2}}\frac{\partial }{\partial r}(r^{2}\frac{\partial c}{\partial r})+kc. \end{array}$$ (3)

for *k* > 0, and *α* > 0. The boundary conditions are

$$\begin{array}{c} \frac{\partial c(0,t)}{\partial r}=0,\;t\geq 0 \end{array}$$ (4)

and

$$\begin{array}{c} c\left(1,t\right)=c_{r_{1}},\;t\geq 0, \end{array}$$ (5)

and the initial concentration distribution is

$$\begin{array}{c} c\left(r,0\right)=c_{t_{0}}\left(r\right),\;0\leq r<1, \end{array}$$ (6)

where *c*_*r*_1__ ≥ 0 and *c*_*t*_0__ > 0. Note that *c*_*r*_1__ ≤ *c*_*t*_0__ for flux toward the exterior of the sphere.

### Analytical solution for the autocatalyst concentration

By substituting *v*(*r*, *t*) = *rc*(*r*, *t*), the partial differential equation (PDE) (Eq 3) and its initial and boundary conditions are transformed to a linear, nonhomogeneous second-order PDE with a source term and nonhomogeneous Dirichlet boundary conditions:

$$\begin{array}{c} \frac{\partial v}{\partial t}=\alpha \frac{\partial^{2}v}{\partial r^{2}}+kv, \end{array}$$ (7)

with boundary conditions

$$\begin{array}{c} v(0,t)=0,\;t\geq 0 \end{array}$$ (8)

and

$$\begin{array}{c} v\left(1,t\right)=c_{r_{1}},\;t\geq 0 \end{array}$$ (9)

and initial condition

$$\begin{array}{c} v\left(r,0\right)=rc_{t_{0}}\left(r\right),\;0\leq r<1. \end{array}$$ (10)

The PDE for *v*(*r*, *t*) can be solved by superposition of the solutions to two related problems [29]: (i) the complementary steady-state boundary value problem,

$$\begin{array}{c} 0=\alpha \frac{d^{2}u}{dr^{2}}+ku \end{array}$$ (11)

with boundary conditions *u*(0) = 0 and *u*(1) = *c*_*r*_1__, where *u*(*r*) is the equilibrium steady-state distribution, and (ii) the PDE for the function *w*(*r*, *t*) ≡ *v*(*r*, *t*) − *u*(*r*),

$$\begin{array}{c} \frac{\partial w}{\partial t}=\alpha \frac{\partial^{2}w}{\partial r^{2}}+kw \end{array}$$ (12)

with homogeneous boundary conditions

$$\begin{array}{c} w(0,t)=w(1,t)=0,\;t\geq 0 \end{array}$$ (13)

and initial condition

$$\begin{array}{c} w\left(r,0\right)=rc_{t_{0}}\left(r\right)-u\left(r\right),\;0<r<1. \end{array}$$ (14)

The steady-state solution, *u*(*r*), and the solution to (Eq 12) for *w*(*r*, *t*) by the method of eigenfunction expansion are derived below and then combined to obtain the solutions for *v*(*r*, *t*) and *c*(*r*, *t*).

**Steady-state solution for** *u*(*r*). The steady-state boundary value problem (Eq 11) can be rewritten as

$$\begin{array}{c} 0=\frac{d^{2}u}{dr^{2}}+\Phi^{2}u,\;0\leq r\leq 1, \end{array}$$ (15)

where the Thiele modulus, Φ, for this first-order reaction-diffusion system is

$$\begin{array}{c} \Phi =\sqrt{k/\alpha }. \end{array}$$ (16)

The Thiele modulus characterizes the relative importance of diffusion and reaction and is defined as the ratio of the characteristic times for diffusion (1/*α*) and reaction in the absence of mass transfer limitations (1/*k*). The solution is of the form [30]

$$\begin{array}{c} u=A\cos(\Phi r)+B\sin(\Phi r). \end{array}$$ (17)

The boundary condition *u*(0) = 0 for finite values of *c* requires that *A* = 0. At the surface,

$$\begin{array}{c} u\left(1\right)=c_{r_{1}}=B\sin\Phi , \end{array}$$ (18)

so *B* = *c*_*r*_1__/ sinΦ. The solution to the steady-state ordinary differential equation (ODE) with nonhomogeneous boundary conditions is

$$\begin{array}{c} u\left(r\right)=rc\left(r\right)=\frac{c_{r_{1}}\sin\left(\Phi r\right)}{\sin\Phi }. \end{array}$$ (19)

**Method of eigenfunction expansion solution for** *w*(*r*, *t*). The linear, nonhomogeneous PDE with homogeneous boundary conditions given by (Eq 12) can be solved using the method of eigenfunction expansion [29], which consists of expanding the unknown solution *w*(*r*, *t*) in a series of the eigenfunctions for the related homogeneous problem:

$$\begin{array}{c} w\left(r,t\right)=\sum_{n=1}^{\infty }a_{n}\left(t\right)\phi_{n}\left(r\right), \end{array}$$ (20)

where *a*_*n*_(*t*) are the time-dependent, generalized Fourier coefficients and *ϕ*_*n*_(*r*) are the eigenfunctions of the homogeneous PDE for diffusion without a source term with homogeneous Dirichlet boundary conditions,

$$\begin{array}{c} \phi_{n}\left(r\right)=\sin(n\pi r). \end{array}$$ (21)

The eigenfunction expansion of the source term is

$$\begin{array}{c} kw\left(r,t\right)=\sum_{n=1}^{\infty }b_{n}\left(t\right)\phi_{n}\left(r\right), \end{array}$$ (22)

where *b*_*n*_(*t*) = *ka*_*n*_(*t*) since the source term is first-order in the linearized concentration.

The Fourier sine series can be differentiated term by term since *w*(*r*, *t*) and sin(*nπr*) satisfy the same boundary conditions [29]. Inserting the eigenfunction expansions for *w*(*r*, *t*) from (Eq 20) and the source term from (Eq 22) into the PDE in (Eq 12) yields

$$\begin{array}{c} \sum_{n=1}^{\infty }\frac{da_{n}}{dt}\phi_{n}=\sum_{n=1}^{\infty }(-\alpha n^{2}\pi^{2})a_{n}\phi_{n}+\sum_{n=1}^{\infty }ka_{n}\phi_{n}. \end{array}$$ (23)

For each *n* = 1, 2, …,

$$\begin{array}{c} \frac{da_{n}}{dt}+(\alpha n^{2}\pi^{2}-k)a_{n}=\frac{da_{n}}{dt}+(n^{2}\pi^{2}-\Phi^{2})\alpha a_{n}=0. \end{array}$$ (24)

The solution to (Eq 24) is

$$\begin{array}{c} a_{n}\left(t\right)=a_{n}\left(0\right)\exp(-(n^{2}\pi^{2}-\Phi^{2})\alpha t), \end{array}$$ (25)

where the *a*_*n*_(0) can be derived by multiplying (Eq 20) by *ϕ*_*m*_ for *t* = 0, considering the orthogonality of the eigenfunctions, and integrating over the spatial domain to give

$$\begin{array}{ccc} a_{n}\left(0\right) & = & \frac{\int_{0}^{1}w\left(r,0\right)\phi_{n}dr}{\int_{0}^{1}\phi_{n}^{2}dr}\\ & = & 2\int_{0}^{1}(v(r,0)-u)\sin(n\pi r)dr\\ & = & \int_{0}^{1}2rc_{t_{0}}\left(r\right)\sin(n\pi r)dr+\frac{2c_{r_{1}}n\pi {\left(-1\right)}^{n}}{n^{2}\pi^{2}-\Phi^{2}} \end{array}$$ (26)

In the specific case of uniform initial distribution, *c*_*t*_0__(*r*) = *c*_*t*_0__ and

$$\begin{array}{c} a_{n}\left(0\right)=\frac{-2c_{t_{0}}{\left(-1\right)}^{n}}{n\pi }+\frac{2c_{r_{1}}n\pi {\left(-1\right)}^{n}}{n^{2}\pi^{2}-\Phi^{2}}. \end{array}$$ (27)

Substituting the expressions for *ϕ*_*n*_ from (Eq 21), *a*_*n*_ from (Eq 25), and *a*_*n*_(0) from (Eq 26) into (Eq 20) yields the solution to the nonhomogeneous PDE with homogeneous boundary conditions (Eq 12):

$$\begin{array}{ccc} w(r,t) & = & \sum_{n=1}^{\infty }(\int_{0}^{1}2rc_{t_{0}}\left(r\right)\sin(n\pi r)dr+\frac{2c_{r_{1}}n\pi {\left(-1\right)}^{n}}{n^{2}\pi^{2}-\Phi^{2}})\\ & & \times \;\exp(-(n^{2}\pi^{2}-\Phi^{2})\alpha t)\sin(n\pi r). \end{array}$$ (28)

**Solution for** *v*(*r*, *t*). Substituting the expressions for *u* from (Eq 19) and *w* from (Eq 28) into *v* = *u*+*w* yields the solution to the nonhomogeneous PDE with nonhomogeneous boundary conditions (Eq 7):

$$\begin{array}{ccc} v(r,t) & = & \frac{c_{r_{1}}\sin\left(\Phi r\right)}{\sin\Phi }\\ & & +\;\sum_{n=1}^{\infty }(\int_{0}^{1}2rc_{t_{0}}\left(r\right)\sin(n\pi r)dr+\frac{2c_{r_{1}}n\pi {\left(-1\right)}^{n}}{n^{2}\pi^{2}-\Phi^{2}})\\ & & \times \;\exp(-(n^{2}\pi^{2}-\Phi^{2})\alpha t)\sin(n\pi r). \end{array}$$ (29)

**Solution for** *c*(*r*, *t*). The concentration of the autocatalyst in radial coordinates is

$$\begin{array}{c} c=\frac{v}{r}. \end{array}$$ (30)

The indeterminate form at *r* = 0 is resolved by

$$\begin{array}{c} \lim_{r\to 0}\frac{\sin(xr)}{r}=\lim_{r\to 0}x\cos\left(xr\right)=x, \end{array}$$ (31)

where *x* denotes a constant. The autocatalyst concentration at *r* = 0 is

$$\begin{array}{ccc} c(0,t) & = & \frac{c_{r_{1}}\Phi }{\sin\Phi }\\ & & +\sum_{n=1}^{\infty }n\pi (\int_{0}^{1}2rc_{t_{0}}\left(r\right)\sin(n\pi r)dr+\frac{2c_{r_{1}}n\pi {\left(-1\right)}^{n}}{n^{2}\pi^{2}-\Phi^{2}})\exp(-(n^{2}\pi^{2}-\Phi^{2})\alpha t), \end{array}$$ (32)

and at 0 < *r* < 1 is

$$\begin{array}{ccc} c(r,t) & = & \frac{c_{r_{1}}\sin\left(\Phi r\right)}{r\sin\Phi }\\ & & +\;\sum_{n=1}^{\infty }(\int_{0}^{1}2rc_{t_{0}}\left(r\right)\sin(n\pi r)dr+\frac{2c_{r_{1}}n\pi {\left(-1\right)}^{n}}{n^{2}\pi^{2}-\Phi^{2}})\\ & & \times \;\exp(-(n^{2}\pi^{2}-\Phi^{2})\alpha t)\frac{\sin(n\pi r)}{r}. \end{array}$$ (33)

With uniform initial distribution, (Eq 32) and (Eq 33) become

$$\begin{array}{ccc} c(0,t) & = & \frac{c_{r_{1}}\Phi }{\sin\Phi }\\ & & +\;2\sum_{n=1}^{\infty }(\frac{c_{r_{1}}n^{2}\pi^{2}}{n^{2}\pi^{2}-\Phi^{2}}-c_{t_{0}}){\left(-1\right)}^{n}\exp(-(n^{2}\pi^{2}-\Phi^{2})\alpha t) \end{array}$$ (34)

and

$$\begin{array}{ccc} c(r,t) & = & \frac{c_{r_{1}}\sin\left(\Phi r\right)}{r\sin\Phi }\\ & & +\;2\sum_{n=1}^{\infty }(\frac{c_{r_{1}}n\pi }{n^{2}\pi^{2}-\Phi^{2}}-\frac{c_{t_{0}}}{n\pi }){\left(-1\right)}^{n}\exp(-(n^{2}\pi^{2}-\Phi^{2})\alpha t)\frac{\sin(n\pi r)}{r}. \end{array}$$ (35)

## Numerical solution for the autocatalyst concentration

The partial differential equation for the autocatalyst concentration Eqs (3)–(6) can also be solved by numerical methods. The method of lines [31, 32] reduces the PDE to a system of ODEs by discretizing the radial dimension onto a finite grid with equal spacing Δ*r* and coordinates *r*_*i*_ = *i*Δ*r* for *i* = 0, 1, …, *N*. Using the boundary conditions and the second-order centered finite difference approximations for spherical coordinates [33] to approximate the spatial derivatives, the semi-discrete ODE for the concentration at each grid point, *c*_*i*_(*t*), is

$$\begin{array}{c} \frac{dc_{i}}{dt}=\left\{ \begin{array}{cc} \frac{6\alpha }{\Delta r^{2}}(c_{1}-c_{0})+kc_{0}, & \text{if}\;i=0;\\ 0 & \text{if}\;i=N;\\ \frac{\alpha }{i\Delta r^{2}}(\left(i+1\right)c_{i+1}-2ic_{i}+\left(i-1\right)c_{i-1})+kc_{i}, & \text{otherwise.} \end{array}\right. \end{array}$$ (36)

The system of ODEs was solved using the function ode45 in MATLAB. The numerical solution gives suitable accuracy when compared with the analytical solution.

## Results and Discussion

### Transient autocatalyst profiles

**Fig 1:**
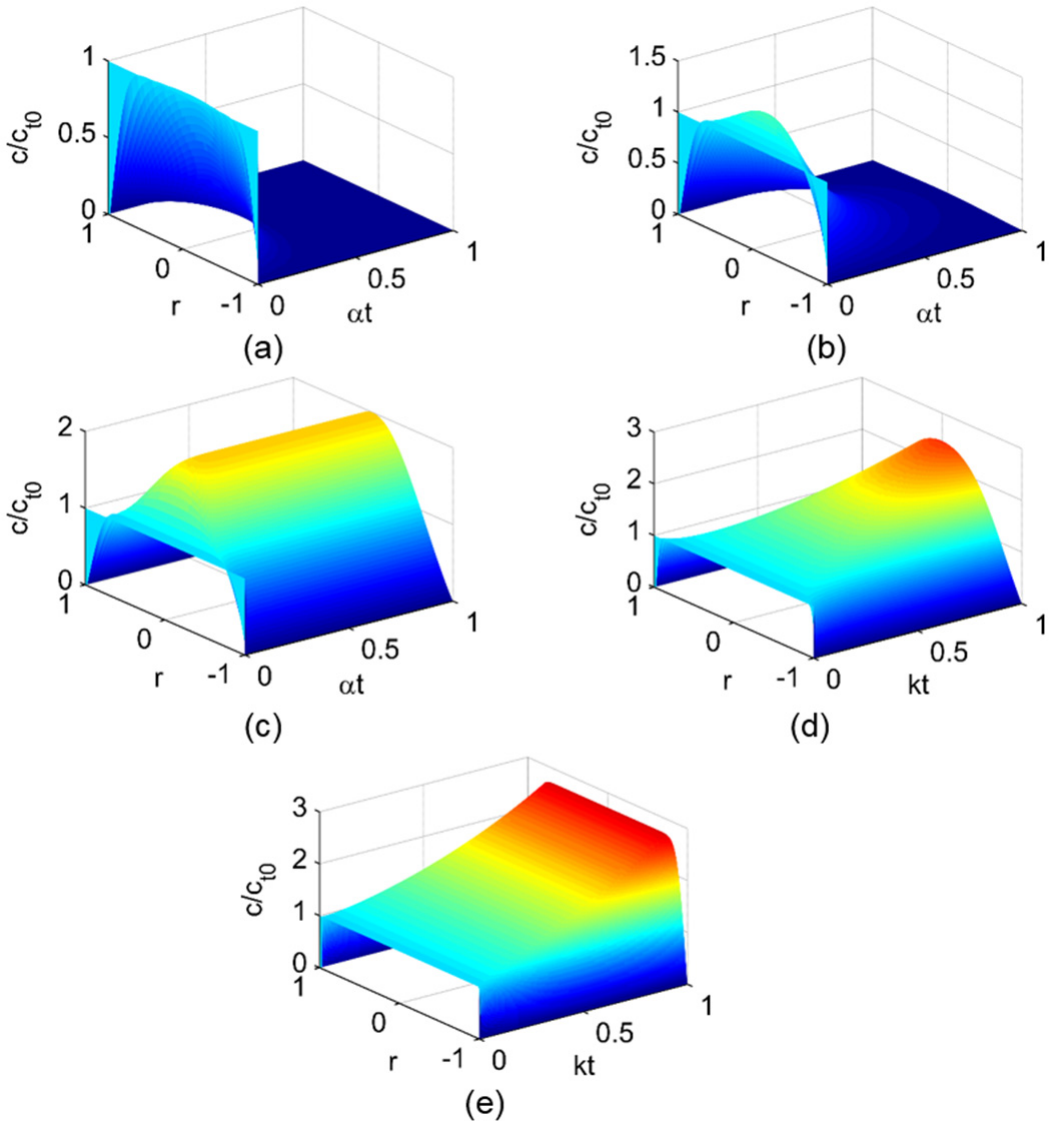
Dimensionless autocatalyst concentration profiles. Autocatalyst concentration *c*/*c*_*t*_0__ profiles as functions of the characteristic time (*αt* for Thiele modulus Φ ≤ *π* and *kt* for Φ > *π*) and dimensionless position *r* for *c*_*r*_1__ = 0: (a) Φ = 0.1*π*, (b) Φ = 0.75*π* (c) Φ = *π*, (d) Φ = 1.25*π*, (e) Φ = 5*π*. The vertical axes of the plots are shown at different scales to zoom in on the full range of change in *c*/*c*_*t*_0__ over the time period. The coloring is consistent between the plots to facilitate comparisons.

**Fig 2:**
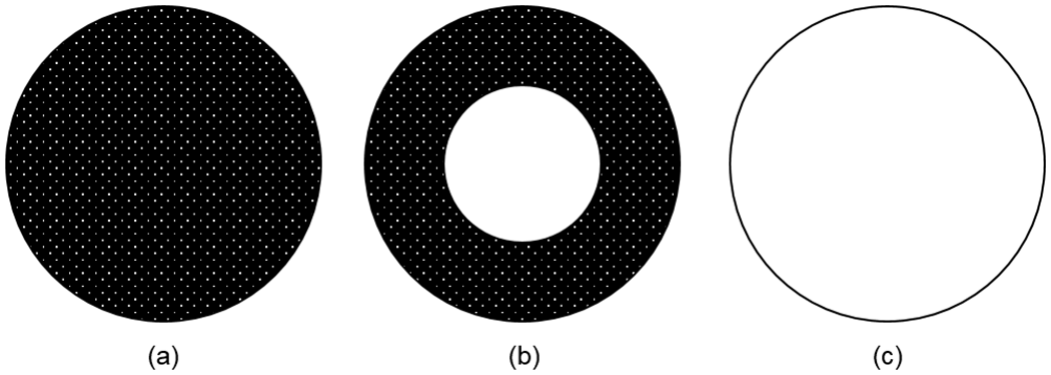
Sketch of spatial distribution of the autocatalyst in microsphere cross-sections in different ranges of Thiele modulus. (a) diffusion throughout for Thiele modulus Φ < < *π*, (b) diffusion near edges and accumulation/enhanced degradation in the center for Φ ≈ *π*, (c) accumulation/enhanced degradation throughout for Φ > > *π*. The autocatalyst is illustrated in white, and the polymer bulk is shown in black.

Our analytical expressions for the transient radial concentration of the autocatalyst Eqs (32)–(35) quantify the importance of the Thiele modulus, Φ, for determining whether PLGA degradation and erosion are enhanced by accumulation or diminished by diffusion. The results presented here are all for the case of uniform initial distribution *c*_*t*_0__(*r*) = *c*_*t*_0__ given by Eqs (34)–(35). For small values of Φ (Fig 1a-1b), diffusion dominates the conservation equation, so any amount of the autocatalyst generated by the reaction diffuses away quickly and a steady state can be reached. Such a microsphere experiences homogeneous erosion without substantially-catalyzed degradation (Fig 2a). Small microspheres, fast diffusion, or slow reaction can give small values of Φ. For intermediate values of Φ on the order of *π* (Fig 1c-1d), the autocatalyst accumulates in the center of the microsphere forming a hollow interior (Fig 2b). For large values of Φ (Fig 1e), the first-order reaction generation dominates the conservation equation, so the autocatalyst accumulates in the interior faster than it can diffuse from the sphere. Autocatalytic degradation is severe throughout the microsphere (Fig 2c), and the entire particle may erode rapidly. The predicted autocatalyst concentration profiles (Fig 1) and the illustrations of the spatial distribution of the autocatalyst in microspheres of different sizes where small sizes correspond to small values of Φ (Fig 2) are consistent with experimental visual evidence of the microspheres-size-dependent spatial distribution of the acidic microclimate within degrading PLGA microspheres [34].

### Transition between regimes

**Fig 3:**
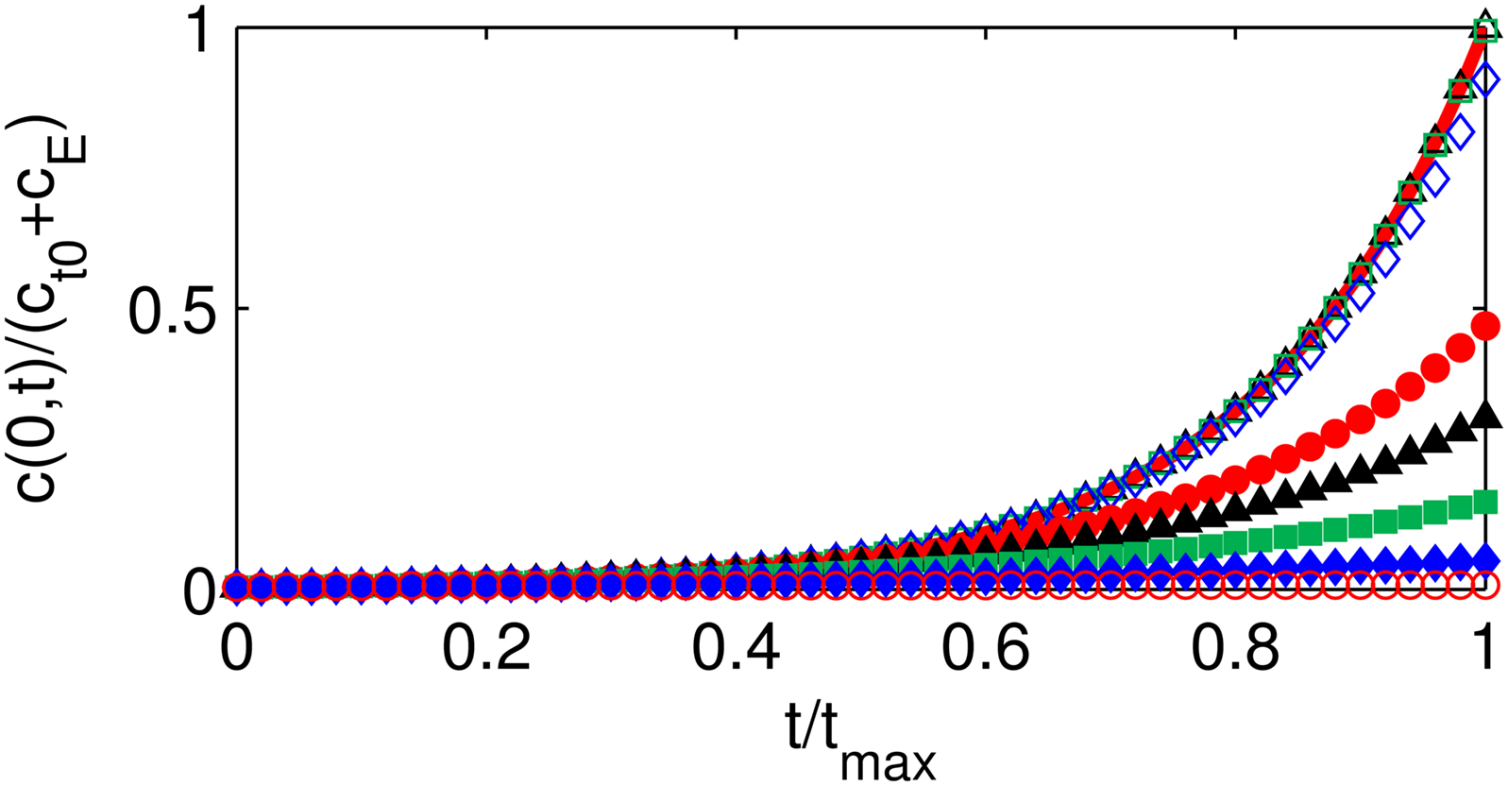
Dimensionless autocatalyst concentration profiles at *r* = 0 for large Thiele modulus values. Autocatalyst concentration *c*/(*c*_*t*_0__+*c*_E_) profiles at *r* = 0 as functions of time scaled by the maximum reaction time *t*_max_ for *c*_*r*_1__ = 0 and large values of Thiele modulus Φ: Φ = *π* (red open circles), Φ = 1.25*π* (blue solid diamonds), Φ = 1.5*π* (green solid squares), Φ = 1.75*π* (black solid triangles), Φ = 2*π* (red solid circles), Φ = 2*π* (blue open diamonds), Φ = 3*π* (green open squares), Φ = 5*π* (black open triangles). The solution approaches the reaction-dominant limit *c*_rxn_ (red solid curve) as Φ → ∞.

**Fig 4:**
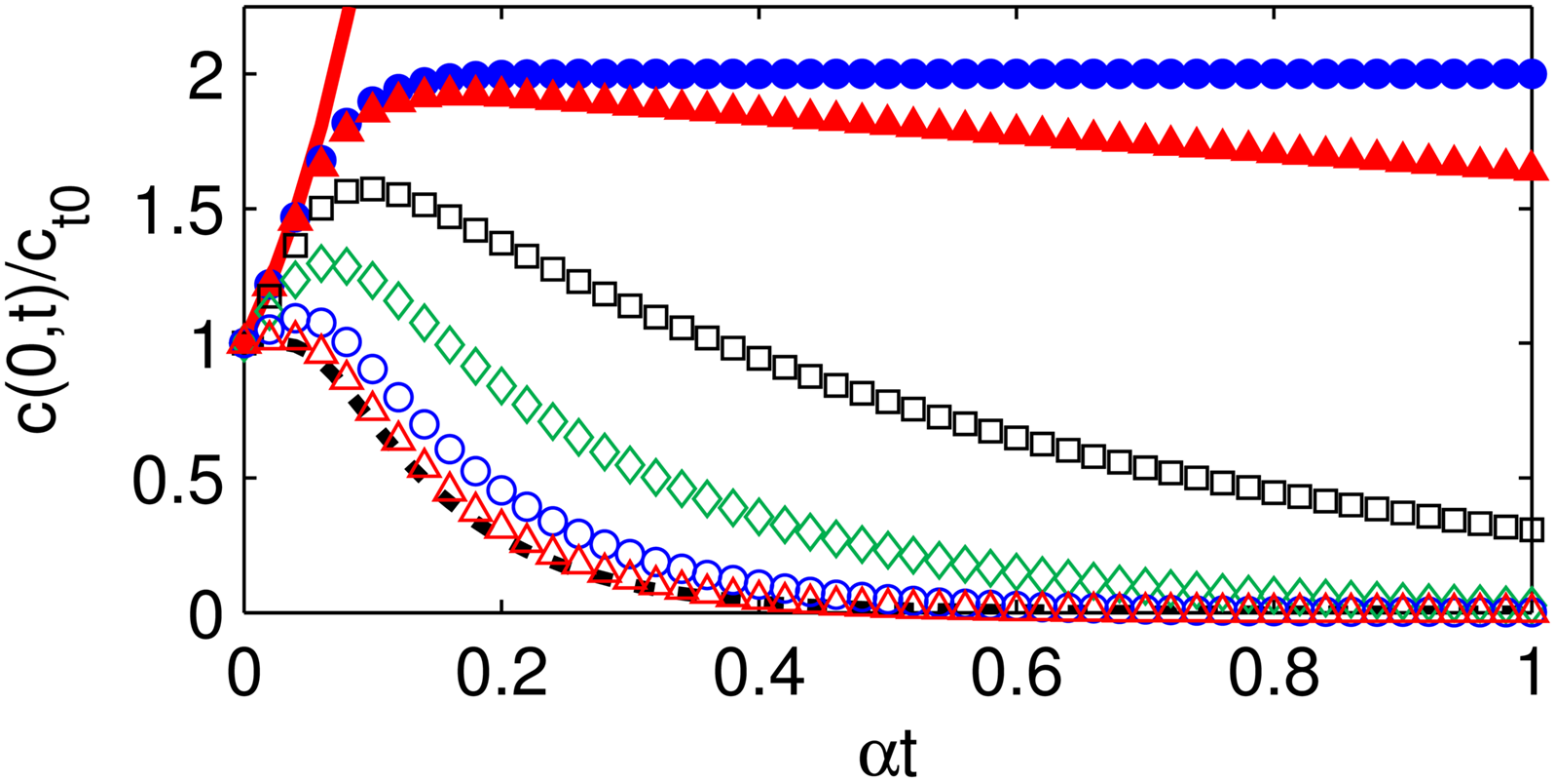
Dimensionless autocatalyst concentration profiles at *r* = 0 for intermediate Thiele modulus values. Autocatalyst concentration *c*/*c*_*t*_0__ profiles at *r* = 0 as functions of the characteristic time for diffusion for *c*_*r*_1__ = 0 and intermediate values of Thiele modulus Φ: Φ = 0.25*π* (red open triangles), Φ = 0.5*π* (blue open circles), Φ = 0.75*π* (green open diamonds), Φ = 0.9*π* (black open squares), Φ = 0.99*π* (red solid triangles), Φ = *π* (blue solid circles). The solutions are bounded by the diffusion-dominant limit *c*_diffn_ (black dotted curve) as Φ → 0 and the reaction-dominant limit *c*_rxn_ (red solid curve) as Φ → ∞.

To assess the bounds on the concentration profiles in different ranges of Φ, the maximum values of the concentration profiles, which occur at the microsphere center *r* = 0, are compared (Figs 3 and 4). The upper and lower bounds are the reaction-dominant limit and the diffusion-dominant limit, respectively.

Considering only the degradation reaction without diffusion, the autocatalyst concentration is described by

$$\begin{array}{c} \frac{\partial c_{\text{rxn}}}{\partial t}=kc_{\text{rxn}}, \end{array}$$ (37)

which has the solution

$$\begin{array}{c} c_{\text{rxn}}\left(r,t\right)=c_{t_{0}}\exp\left(kt\right), \end{array}$$ (38)

where *c*_rxn_ is referred to as the reaction-dominant limit. This limit is the commonly treated case for autocatalytic hydrolysis kinetics [13], where the concentration at each position is independent of its neighbors. As Φ → ∞, *c*(*r*, *t*) → *c*_rxn_(*t*) (Fig 3), so values in the range Φ ≥ *π* are in the erosion-controlled regime.

In a PLGA microsphere, the degradation reaction cannot continue infinitely because the hydrolysis reaction only proceeds as long as ester bonds can be cleaved in the polymer. The maximum time for the reaction occurs when all of the ester bonds have been converted to monomers, so *c*_rxn_(*r*, *t*_max_) = *c*_*t*_0__ + *c*_E_ and

$$\begin{array}{c} t_{\max}=\frac{1}{k}\ln\frac{c_{t_{0}}+c_{E}}{c_{t_{0}}}=\frac{1}{k}\ln\frac{M_{n_{0}}}{M_{1}}, \end{array}$$ (39)

where *M*_*n*_0__ is the initial number-average molecular weight and *M*_1_ is the average weight of a monomer. As *t* → *t*_max_ the assumption of constant ester bond concentration is violated. At *t* = 1/*k*, *t*/*t*_max_ ≈ 1–2% for PLGA 50:50 with *M*_1_ = 65.05 Da, *M*_*n*_0__ = 10–1000 kDa, and *k* = 0.012–0.08 day^−1^ [7], thus the constant ester concentration assumption is valid within the characteristic time for the reaction (*kt* = 1 gives *t* ≈ 12–80 days).

Considering only diffusion without the degradation reaction, the autocatalyst concentration is described by

$$\begin{array}{c} \frac{\partial c_{\text{diffn}}}{\partial t}=\alpha \frac{\partial^{2}c_{\text{diffn}}}{\partial r^{2}}, \end{array}$$ (40)

with boundary and initial conditions given by Eqs (5)–(6), which has the solution [33] for *r* = 0

$$\begin{array}{c} c_{\text{diffn}}\left(0,t\right)=c_{r_{1}}+2(c_{r_{1}}-c_{t_{0}})\sum_{n=1}^{\infty }{\left(-1\right)}^{n}\exp(-n^{2}\pi^{2}\alpha t) \end{array}$$ (41)

and for 0 < *r* < 1

$$\begin{array}{c} c_{\text{diffn}}\left(r,t\right)=c_{r_{1}}+\frac{2(c_{r_{1}}-c_{t_{0}})}{\pi r}\sum_{n=1}^{\infty }\frac{{\left(-1\right)}^{n}}{n}\exp(-n^{2}\pi^{2}\alpha t)\sin\left(n\pi r\right), \end{array}$$ (42)

where *c*_diffn_ is referred to as the diffusion-dominant limit. As Φ → 0, *c*(*r*, *t*) → *c*_diffn_(*r*, *t*) (Fig 4), so values in the range Φ < *π* are in the diffusion-controlled regime. For intermediate values of Φ, the autocatalyst concentration is bounded by the diffusion- and reaction-dominant limits; the concentration peaks due to early accumulation and enhanced degradation in the center and later decays due to diffusion. Φ = *π* is the tipping point between the diffusion- and erosion-controlled regimes characterized by exponential decay and exponential growth of the autocatalyst, respectively.

### Steady-state autocatalyst profiles

The autocatalyst concentration, *c*(*r*, *t*), exhibits exponential growth due to the first-order hydrolysis reaction and exponential decay due to diffusion. The steady-state limit is approached as the rates of diffusion and generation by reaction offset each other or when the reacting species has diffused out of the system completely. For the concentration to reach a steady state, the time derivative of *c*(*r*, *t*) must be zero; either the exponential term must (i) be constant or (ii) approach 0 as *t* → ∞. Therefore,

$$\begin{array}{c} n^{2}\pi^{2}-\Phi^{2}\geq 0,\;n=1,2,\ldots . \end{array}$$ (43)

In the most restrictive case of *n* = 1, Φ ≤ *π*.

**Fig 5:**
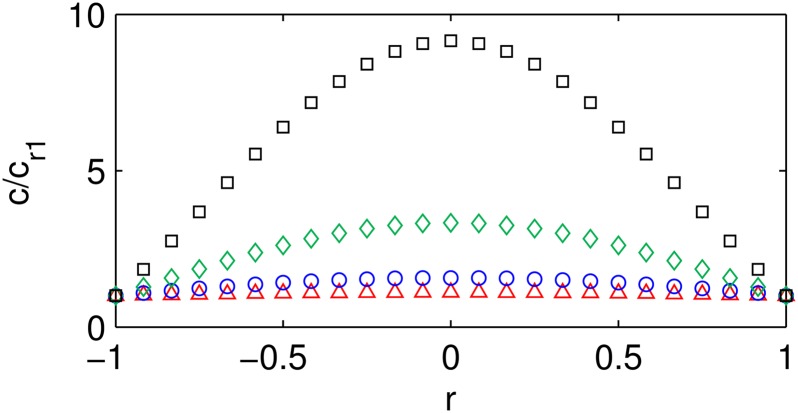
Steady-state dimensionless autocatalyst concentration profiles. Steady-state dimensionless autocatalyst concentration $\underset{t\to \infty }{\lim}c(r,t)/c_{r_{1}}$ profiles as functions of dimensionless position *r* for boundary condition *c*_*r*_1__ > 0 and Thiele modulus Φ < *π*: Φ = 0.25*π* (red open triangles), Φ = 0.5*π* (blue open circles), Φ = 0.75*π* (green open diamonds), Φ = 0.9*π* (black open squares). At *r* = 0, *c*/*c*_*r*_1__ → ∞ as Φ → *π*.

The constant surface boundary condition *c*_*r*_1__ determines the magnitude of the diffusion driving force and indicates the concentration of species in the medium assuming no mass transfer limitations at the surface; this is consistent with a buffered medium where the autocatalyst released from the microsphere is perfectly absorbed by the medium. If *c*_*r*_1__ = 0, values of Φ < *π* have trivial steady-state solutions *c*(*r*, *t*)/*c*_*t*_0__ → 0 as *t* → ∞ where the species diffuses completely out of the sphere preventing runaway of the first-order, autocatalytic reaction, while Φ = *π* has a stable nontrivial solution *c*(*r*, *t*)/*c*_*t*_0__ → 2 as *t* → ∞, balancing the contributions from the reaction and diffusion. If *c*_*r*_1__ > 0, the steady-state solutions for Φ = *mπ* are nontrivial (Fig 5), and neither the steady-state linearized solution, *u*, nor the analytical solution, *c*, are defined for Φ = *mπ*, *m* = 1, 2, …. The transient profiles for Φ = *mπ* can be approximated by the numerical solution even though steady-state solutions do not exist in these cases. The case of *c*_*r*_1__ > 0 is interesting from a mathematical perspective but may only be physically appropriate for media that have high concentrations of strong acid that augment the carboxylic acid end groups in catalyzing the degradation. The boundary condition *c*_*r*_1__ = 0 is valid assuming no autocatalyst in a weakly acidic buffered medium and is used for the results shown in Figs 1, 3, and 4.

## Conclusions

An analytical expression was derived for the transient, radial concentration of a species undergoing simultaneous diffusion and first-order reaction generation with constant, but not necessarily zero, surface boundary concentration. The expression differs from the common reaction-diffusion case treated in the spherical catalyst literature as we treat a generation rather than consumption reaction term. To our knowledge, this is the first application of such an analytical expression for the reaction-diffusion equation to PLGA microspheres to model degradation and erosion of the polymer. Treating the diffusive transport of the autocatalytic species to capture spatial heterogeneities during degradation is a unique contribution of this work.

A limitation of the analytical expression for the autocatalyst concentration is the assumption that the diffusion coefficient is independent of the lengths of the polymer chains to which the autocatalytic carboxylic acid end groups are attached. In reality only a fraction of the polymer chains are small enough to be water-soluble and able to diffuse through the aqueous pores. To account for the overestimation of autocatalyst mobility in the assumption needed to simplify the mathematical formulation, a smaller value for the diffusion coefficient than the value for just the soluble chains should be used to represent the average diffusivity of soluble and insoluble polymer chains. A more detailed model distinguishing between the diffusivities of soluble and insoluble autocatalyst populations would require a numerical solution to the PDE for each population.

The analytical expression for autocatalyst concentration indicates that the Thiele modulus is a key parameter for predicting the transition between the diffusion- and erosion-controlled release regimes. With the coupling between reaction and diffusion of the autocatalyst treated by this model, size-dependent effects on autocatalysis can be explored and incorporated into detailed predictive models for drug delivery from autocatalytic PLGA microspheres, which could ultimately contribute to the *in silico* optimal design of controlled drug release particles.
